# The Unique, the Known, and the Unknown of Spumaretrovirus Assembly

**DOI:** 10.3390/v13010105

**Published:** 2021-01-13

**Authors:** Dirk Lindemann, Sylvia Hütter, Guochao Wei, Martin Löchelt

**Affiliations:** 1 Institute of Virology, Medical Faculty “Carl Gustav Carus”, Technische Universität Dresden, 01307 Dresden, Germany; sylvia.huetter86@gmail.com; 2 CRTD/DFG-Center for Regenerative Therapies, Technische Universität Dresden, 01307 Dresden, Germany; 3 Division Viral Transformation Mechanisms, Research Focus Infection, Inflammation and Cancer, German Cancer Research Center (Deutsches Krebsforschungszentrum, DKFZ), 69120 Heidelberg, Germany; guochao.wei@cuanschutz.edu

**Keywords:** foamy virus, spumavirus, assembly, Env leader protein, particle budding, Pol protein packaging, RNA-mediated Pol tethering, retrovirus evolution, *Ortervirales*

## Abstract

Within the family of *Retroviridae*, foamy viruses (FVs) are unique and unconventional with respect to many aspects in their molecular biology, including assembly and release of enveloped viral particles. Both components of the minimal assembly and release machinery, Gag and Env, display significant differences in their molecular structures and functions compared to the other retroviruses. This led to the placement of FVs into a separate subfamily, the *Spumaretrovirinae*. Here, we describe the molecular differences in FV Gag and Env, as well as Pol, which is translated as a separate protein and not in an orthoretroviral manner as a Gag-Pol fusion protein. This feature further complicates FV assembly since a specialized Pol encapsidation strategy via a tripartite Gag-genome–Pol complex is used. We try to relate the different features and specific interaction patterns of the FV Gag, Pol, and Env proteins in order to develop a comprehensive and dynamic picture of particle assembly and release, but also other features that are indirectly affected. Since FVs are at the root of the retrovirus tree, we aim at dissecting the unique/specialized features from those shared among the *Spuma-* and *Orthoretrovirinae.* Such analyses may shed light on the evolution and characteristics of virus envelopment since related viruses within the *Ortervirales*, for instance LTR retrotransposons, are characterized by different levels of envelopment, thus affecting the capacity for intercellular transmission.

## 1. Introduction

In this review, we attempt to provide an up-to-date summary of our current knowledge about the assembly strategy of a small and distinct group of retroviruses (RVs), the spuma- or foamy viruses (FVs), as well as identifying gaps in our knowledge that require further research. Several unique or at least uncommon features of the molecular biology of currently circulating FVs, which also partly exist in ancient, endogenized FVs, have led to their classification into the distinct subfamily of *Spumaretrovirinae* with only few genera within the family of *Retroviridae* [1]. The rest of the RVs, all members of the *Orthoretrovirinae* subfamily, follow the canonical, orthodox pathway of what is generally assumed or known to characterize RVs in many aspects [2]. The true FV-specific features relate to several aspects of their molecular biology, including defined mechanisms and unique features of particle formation and assembly and are discussed in this review. Really FV-specific features not known for any other RV relate to the:

(i) Genome coding strategy;
(ii) Processing and numbers of particle-associated polymerase Pol and envelope Env proteins;
(iii) Sequence features and the mode and timing of Gag polyprotein processing;
(iv) The packaging and activation of the Pol gene-associated enzymatic functions [3,4].

**Figure 1:**
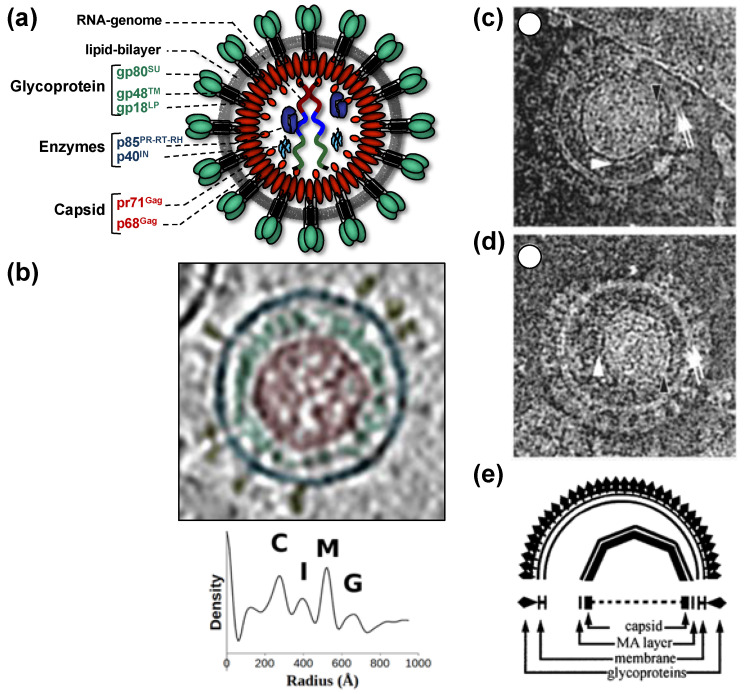
Structure of foamy virus (FV) virions. (**a**) Schematic representation of the prototype FV isolate (PFV) particle structure. pr: precursor protein; p: protein; gp: glycoprotein; (**b**) cryo-electron tomography of PFV virion and the corresponding radial density profile with the various peaks corresponding to the capsid (C), intermediate shell (I), viral membrane (M), glycoprotein (G), labeled and colored in red, green, blue, and yellow respectively. (**c**–**e**) Ultrastructure of feline FV (FFV) virions by cryo-electron microscopy analysis. The MA layer (white arrowheads) follows the shape of the capsid in particles with central (**c**) and off-center (**d**) capsids as schematically shown in panel (**e**). Many particles in the population show internal angular capsid (the edge of the capsid is marked by black arrowheads) displaced from the center of the particle. Panel (**a**) adapted from [3,8]; panel (**b**) adapted from [7]; panel (**c**–**e**) adapted from [6].

All of this leads to the release of virus particles with a unique and characteristic morphology that even allows proper classification via thin-section electron microscopy [5]. Re-evaluation of FV morphology via novel imaging technologies has led to the identification of a novel structural feature amongst RV particles, the matrix (MA) layer or intermediate shell (Figure 1) that follows the virus membrane at budding and subsequently relocates to the capsid’s edges [6,7]. The MA layer is visible evidence that FV assembly, release and maturation is different from that of the other RVs. Besides these features, FV assembly is characterized by the utilization of pathways and mechanisms rarely found in other RVs, for instance, the cytoplasmic pre-assembly of capsids and their subsequent envelopment, budding, and release at the plasma membrane or internal membranes [5].

The utilization of several unique and non-canonical pathways connects to the fact that FVs are the most ancient RVs according to genetic analyses of fossilized endogenous FV (EndFV) genomes preserved in members of all vertebrate branches (classes) from fish to mammals [9,10,11]. FVs combine a deep-rooted, ancient evolutionary history with an almost complete co-speciation with their authentic host and host clades (simians, cattle, cat, equines, and bats) and a high genome conservation possibly due to a tightly cell-associated transmission and a persistent/latent life cycle [4,12,13]. These features may allow us to track the development and evolution of their unique molecular biology and to identify or at least postulate mechanisms that (may) have led to this mosaic of unique and unconventional features. Unravelling the basic mechanisms of FV biology has already opened new avenues of FV vector development, but may also shed new light into the evolution of their apparent apathogenicity and peaceful co-evolution/co-habitation with their hosts [14,15,16].

In this review, we follow the path of FV capsid assembly to the release of the infectious FV particle with its unique morphology. We first describe the viral components of capsid assembly and protein/genome packaging. In the two following sections, processes and mechanisms related to cytosolic capsid assembly and the subsequent transfer to membranes, envelopment, and release are presented. We also identify important gaps in our knowledge worth investigating in the future. All chapters are predominantly based on data from the prototype FV (PFV), which is the end-product of the zoonotic transmission of a chimpanzee FV to an East-African nasopharynx carcinoma patient [17,18,19]. Studies on feline and bovine FV (FFV and BFV) complement and extend these data, allowing the definition of conserved or deviant strategies within the FV subfamily. Finally, we speculate on the interrelationship of the unique or uncommon features and mechanisms of FV assembly and try to link them to FV biology, their application in translational science and the evolution of RV assembly and release.

## 2. The Viral Machinery and Cellular Partners of FV Particle Formation, Envelopment, and Release

### 2.1. FV Gag Proteins

**Figure 2:**
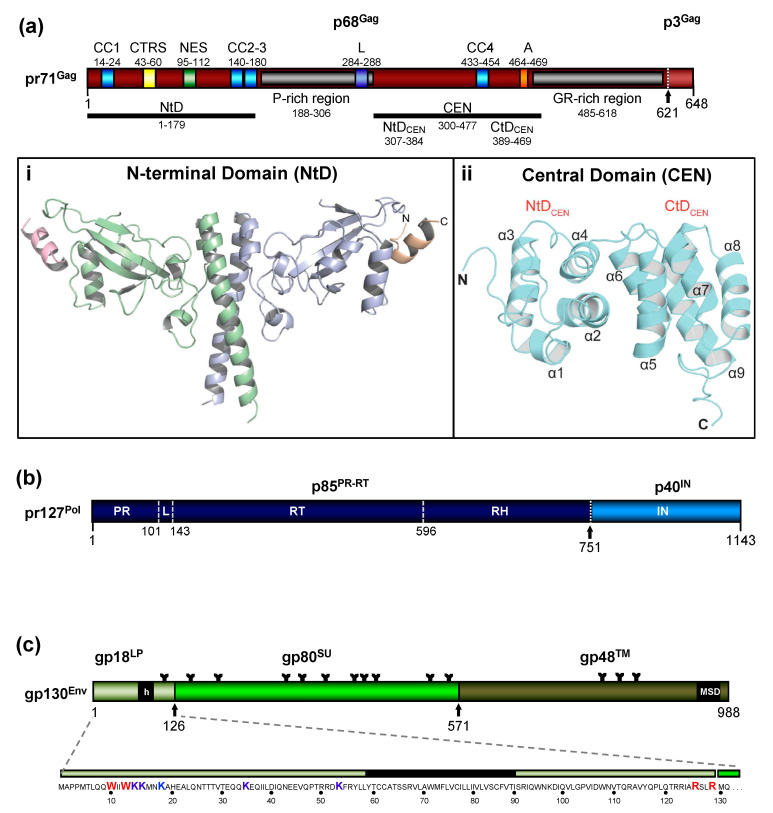
Schematic representation of the FV Gag, Pol, and Env protein organization. (**a**) Schematic illustration of the PFV Gag protein organization and selected functional motifs. Several functional motifs of PFV Gag are highlighted in differently colored boxes. Numbers indicate amino acid (aa) positions of the PFV Gag protein. The black arrow marks the cleavage site of pr71^Gag^ for processing into p68^Gag^ and p3^Gag^. Gray boxes represent the proline-rich (PR-rich) and glycine-arginine-rich (GR-rich) regions respectively. CC1 to CC4: coiled-coil domain 1 to 4; CTRS: cytoplasmic targeting and retention signal; NES: nuclear export sequence; L: late budding domain motif; A: assembly motif. (**i**) Cartoon representation of the three-dimensional (3D) structure of the PFV Gag N-terminal domain (NtD, aa 1–179) homodimer in complex with Env leader protein (Elp/LP) peptides. The Gag-NtD monomer-A is shown in pale blue and monomer-B in green. The helical Elp/LP peptides bound at the periphery of each head domain are colored magenta and gold with N and C termini indicated (**ii**) Cartoon representation of the 3D structure of the PFV-Gag central domain (CEN, aa 300–477) with its two subdomains NtD_CEN_ and CtD_CEN_. The peptide backbone is shown in cyan. The secondary structure elements are numbered sequentially from the amino-terminus and the N and C termini are indicated. Helices α1 to α4 and α5 to α9 that comprise NtD_CEN_ and CtD_CEN_, respectively, are indicated. (**b**) Schematic illustration of the PFV Pol protein organization. Numbers indicate aa positions of the PFV Pol protein. The black arrow marks the cleavage site of pr127^Pol^ for processing into p85^PR-RT-RH^ and p40^IN^. PR: protease domain; L: linker sequence; RT: reverse transcriptase domain; RH: RNase H domain; IN: integrase domain. (**c**) Schematic organization of PFV Env protein. The furin cleavage sites within the gp130^Env^ precursor that are used for generation of the mature gp18^LP^, gp80^SU^, and gp48^TM^ subunits are indicated by arrows. The individual subunits are shown as boxes in different shades of green. Hydrophobic sequences spanning the membrane in Elp/LP (h) and transmembrane (TM) (membrane-spanning domain, MSD) subunit are indicated. The aa sequence of the PFV Env Elp/LP subunit is shown in the enlargement below. The conserved WxxW and RxxR motif are highlighted in red, the lysine residues potentially ubiquitinated are highlighted in blue. The approximate positions of PFV Env N-glycosylation sites are marked by Y-shaped symbols. Panel (**a**,**b**,**c**) adapted from [3,8,22]; panel (**ai**) adapted from [25]; panel (**aii**) adapted from [24].

The FV Gag proteins are unique among RVs due to a strongly restricted proteolytic processing as the mature retroviral matrix (MA), capsid (CA), and nucleocapsid (NC) proteins may be only generated during particle disassembly in the newly-infected cell [20,21,22]. A consistent and functionally relevant proteolytic processing site is located about 30 amino acids (aa) upstream of the C terminus of the 52 to 71 kDa Gag precursor (Figure 2a) [20,23]. It is not required for the release of morphologically deviant particles but it is essential for particles gaining full infectivity. The potential function of the terminal 3–4 kDa C-terminal Gag peptide in the Gag precursor and its fate, localization, and function after proteolytic release by the FV protease (PR) are unknown, but of high scientific interest.

Due to the very limited processing of FV Gag and the lack of sequence and structural homology to the orthoretroviral MA and NC proteins ([20,24] and see below), nomenclature of the functional domains of FV Gag is challenging and may need revision. Until more structural data on FV Gag proteins and FV capsids become available, we propose to use the current nomenclature of retroviral Gag domains (MA, CA, NC) as an interim solution: the overall spatial domain organization is retained in FV Gag and several functions are conserved but probably have different evolutionary roots (see below). However, to reflect the lack of conventional Gag processing, we propose adding the term domain as follows: MA-domain, CA-domain, and NC-domain.

The FV Gag proteins lack the canonical major homology region (MHR) in the CA-domain and the Cys-His fingers in the NC-domain [23]. The approximately 20 aa-long Gag MHR of orthoretroviruses is required for proper particle/capsid assembly, while one or two Cys-His fingers are essential for RNA binding and viral genome encapsidation [5]. A MHR-corresponding element has not been found in FV Gag, however, the glycine- and arginine-rich (GR) sequences in the NC-domain may be the functional counterpart for nucleic acid binding conferred by the Cys-His fingers in the orthoretroviruses (Figure 2a). In primate FVs, the GR-rich NC-domain is organized in three 11 to 13 aa-long motifs (GR boxes I to III, [26]) which is not the case in the other known FVs including the ancient EndFVs [4,23]. In addition, an N-terminal Gag myristoylation signal and/or hints for Gag acetylation are not present in the N terminus of Gag, an additional distinguishing feature of FVs.

When comparing different members of orthoretrovirus genera, the Gag protein sequence has a higher conservation than Env, an observation that reflects the naming of Gag as the group-specific antigen [5]. However, this is not the case for FVs where Env proteins of different FV genera show higher degrees of homology/similarity than Gag [27]. Gag proteins of different FVs genera also vary considerably in size (between about 510 to 650 aa residues, [28]). This size variation is mainly due to a proline-rich and thus highly flexible region, which is flanked at its N terminus by a domain corresponding to the MA protein of other RVs, and at its C terminus by the capsid-forming and well-conserved CA-domain (Figure 2a) [23,28]. Since the FV MA- and this proline-rich domain are not separated by proteolytic processing in released particles, differences in the width of the MA layer formed by these N-terminal elements of FFV Gag are visible in cryo electron micrographs (Figure 1) [6,7].

### 2.2. FV Pol Proteins

**Figure 3:**
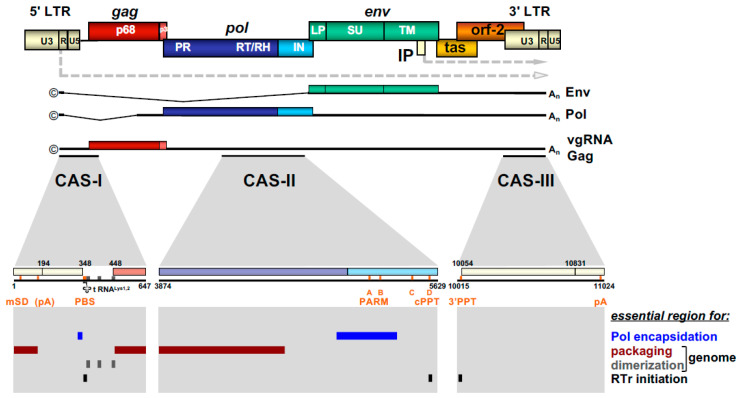
FV provirus organization, genomic and structural gene transcripts, and essential cis-acting viral RNA sequence elements. Schematic illustration of the PFV proviral DNA genome structure with long terminal repeats (LTRs) and open reading frames (ORFs) indicated as boxes. For ORFs encoding Gag, Pol, and Env precursor proteins, the regions encompassing the mature subunits generated by proteolytic processing are indicated by different colors and are labeled accordingly. Transcription initiation sites in the LTR and internal promoter (IP) and direction of transcription are indicated by dashed line arrows. Spliced and unspliced viral transcripts originating from the LTR and encompassing the viral RNA genome (vgRNA) or encoding the structural proteins Gag, Pol, and Env are schematically illustrated below and their respective coding capacity indicated to the right. Transcripts originating at the IP are omitted. Cis-acting sequence (CAS) elements localized within the full-length viral RNA genome, which are essential for viral replication, are indicated by black bars underneath the vgRNA. Individual functionally important or essential RNA sequence motifs are marked in the enlarged individual CAS elements below. Numbers represent nucleotide positions of the viral PFV RNA genome (HSRV2 isolate). At the bottom, individual regions within the vgRNA, which are essential for specific functions in viral replication as indicated to the right, are marked as differentially colored bars. U3: unique 3′ LTR region; R: repeat LTR region; U5: unique 5′ LTR region; ©: cap structure; A_n_: poly A tail; mSD: major splice donor; PBS: primer binding site; PARM: protease activating RNA motif; cPPT: central poly-purine tract; 3′ PPT: 3′ poly-purine tract; pA: polyadenylation signal; A-D: purine-rich sequence motifs PPT A through D; RTr: reverse transcription; tas: transactivator of spumaviruses; bel-2: between envelope and LTR ORF-2. Adapted from [8].

The most obvious unique feature of FV Pol is the fact that it is not expressed as a Gag-Pol fusion protein [29,30]. Instead, it is translated from a spliced, sub-genomic *pol* transcript as a separate Pol protein independent of Gag [27,31,32,33] (Figure 3). The level of the Pol mRNA appears to be regulated by use of a suboptimal splice site [34]. However, in BFV, the spliced FV pol mRNA has been found to have similar levels compared to full-length RNA [27], which may result in substantial Pol protein levels. This unique situation is very likely to affect several “downstream” features of the different *pol*-encoded proteins, but also of the Pol polyprotein precursor, for instance, processing and activation/regulation of the individual enzymatic functions with only vaguely known secondary effects concerning RT and PR activation. Most importantly, Pol is only processed into a PR-RT-RH “polyprotein” and the mature integrase (IN) [35] (Figure 2b). The absence of a free PR protein within released virus particles and newly infected cells may be one of the consequences of this unique translation/expression strategy (via a spliced transcript) as a means to control unscheduled and premature PR activation. Some unique or deviant sequence features of Pol proteins, for instance the unusual catalytic center of PR or the comparative ease of obtaining integrase (IN) crystals and thus the first authentic structural insights into RV/retroid element IN structure and function [20,36] may be consequences of the unique Pol expression strategy and its molecular features. Additionally, a “passive” co-packaging of Pol as a Gag-Pol protein during capsid assembly is not possible during FV assembly as discussed below (see Section 3.2).

### 2.3. FV Env Proteins

In contrast to Pol, the transcription and endoplasmatic reticulum (ER) membrane-targeted translation of FV Env follow the general strategy of RV gene expression via a family of singly or doubly spliced transcripts with small non-coding upstream exons and/or differences in the untranslated region upstream of a unique start codon [37] (Figure 3). However, co- and posttranslational processing of FV Env contains several unique aspects amongst RVs.

The organization of the FV Env precursor follows the domain structure of retroviral Env proteins and the peptide backbone of its surface (SU) and transmembrane (TM) regions comprise similar numbers of aa [5] (Figure 2c). The N-terminal signal- (SPs) or leader peptides (LPs) of orthoretroviral Env proteins, which are needed for ER-targeted biosynthesis and concomitant translocation of most of Env into the ER lumen, are usually small (<35 aa residues) and co-translationally processed by signal peptidase(s) (SPase). They are not a component of the mature, oligomeric retroviral glycoprotein complex (GPC) of virus particles, and are rapidly degraded. None of this is the case for FVs [3,5,38].

In FV glycoproteins, the functional homologue of the LP (for ER targeting) is embedded in a domain/subunit of about 120 to 130 aa length (Figure 2c). This glycoprotein subunit, designated either Elp for Env leader peptide or just LP for leader peptide, is derived from the N terminus of the Env precursor mainly via posttranslational processing by furin or furin-like cellular proteases during intracellular Env precursor trafficking [39,40]. FV Elp/LP has a type II membrane topology and is a stable and abundant component of released and infectious FV particles [41,42] (Figure 1a and Figure 2c). Its N-terminal, cytoplasmic domain (CyD) of about 60 aa in size, is followed by a standard hydrophobic membrane-spanning domain (MSD), and a short, about 40 aa-long C-terminal extracellular domain. In addition to processing at the two furin-cleavage sites, one between Elp/LP and SU and the other separating SU from TM, the PFV (gp130^Env^) and FFV Env precursors and possibly Elp/LP as well, are substrates for SPase and signal peptide peptidase-like (SPPL) proteases [39,42,43]. SPPL2a/b appears to process only PFV gp18^LP^ whereas SPPL3 processes both PFV gp18^LP^ and gp130^Env^ within the MSD of the Elp/LP domain, although the exact cleavage site(s) could not be identified. Whether SPase- and SPPL-mediated cleavage of FV Env is of functional relevance for viral replication or just involved in cellular degradation of Elp/LP, and whether SPPL-mediated cleavage products are an integral component of released FV virions, has not been investigated [43]. If they are not just intermediate products prone for final degradation, these SPPL-derived Elp/LP processing products may possess essential functions in the FV replication cycle.

Strikingly, *env* open reading frame (ORF) encoded LPs of other exogenous (mouse mammary tumor virus, MMTV, and Jaagsiekte sheep retrovirus, JSRV) or endogenous RVs (human endogenous retrovirus K, HERV-K) have reported functions as nuclear export factors of not fully spliced viral mRNAs [44,45,46,47,48]. In case of MMTV and HERV-K, these *env* ORF-encoded LP can be derived from both the Env precursor and separate nuclear export factors (Rem, Rec), translated from alternatively spliced Env mRNAs [44,48,49], whereas in case of JSRV, a prematurely polyadenylated Env mRNA variant has been reported as additional source for Env LP [46]. Interestingly, for their nuclear export function, these retroviral *env* ORF encoded-LPs require extraction from the ER membrane prior to nuclear localization. In case of MMTV Env LP, this involves a new retro-translocation mechanism [50]. Perhaps FV Elp/LP, and in particular its SPPL-derived processing products may extract from cellular membranes and have similar yet undiscovered function in FV RNA export.

Like other retroviruses, FV Env is not only known to be modified by proteolytic processing, but also undergoes additional posttranslational modifications. Similarly to orthoretroviral Env proteins, FV Env is heavily glycosylated at 14 of 15 N-glycosylation sites [51] (Figure 2c). However, only three sites (N8 in SU and N13, N15 in TM) appear to be individually essential for virus morphogenesis. Absence of N-glycosylation at either of these sites results in intracellular transport defects of the respective mutant, putatively due to glycoprotein misfolding and abolished Env-dependent particle release. Whether FV Env proteins also contain O-linked sugars, such as some other retroviral Env proteins, has not yet been investigated.

Among retroviral glycoproteins, another posttranslational modification is unique [52]. The Elp/LP subunit of primate FV Env proteins is ubiquitinated at several lysine residues located in its N-terminal CyD (Figure 2c). Primate Elp/LP ubiquitination appears to reduce cell surface glycoprotein abundance and suppresses the intrinsic capacity of primate FV glycoproteins to promote release of capsid-less, subviral particles (SVPs) [52,53]. Analyses in FFV do not reveal indications of Elp/LP myristoylation and the two lysine residues implicated in the regulation of PFV particle release via ubiquitination are conserved only among SFVs [39,42]. In addition, a di-lysine ER retrieval signal is present in most known FVs except EFV and BFV [37]. It would be interesting to determine whether different pathways co-exist to regulate Elp/LP functions and particle release in individual FV groups.

As discussed in more detail below, the intact FV Env protein is required for FV particle budding. In particular, a pair of tryptophan residues close to the N terminus of Elp/LP have been shown to be absolutely required for FV budding and infectivity due to specific interactions with N-terminal Gag (MA-domain) residues (Figure 2c). This finding further supports the special role of FV Env as “more than just needed for targeting and entering the host cell”, which may be the case for most orthoretroviruses.

### 2.4. FV RNA Genome 

The retroviral RNA genome is characterized by specific folding resulting in secondary and tertiary structures, which allow its interaction with specific protein effectors, for instance for nucleo-cytoplasmic transport, but most importantly, for specific particle packaging or encapsidation [5]. The Psi genome packaging element on the full-length RNA is usually located in the 5′ part of the genome. In most cases, it is located in the untranslated region upstream of the *gag* ORF and possibly extends further into coding sequences [5]. This feature is also shared by FVs and the respective genomic element is designated the cis-acting sequence I (CAS-I) (Figure 3). However, FVs require another cis-acting genomic RNA element, CAS-II, for viral infectivity and FV vector function [54,55,56,57]. CAS-II is reported to harbor in its 5′ part additional RNA packaging elements, while its 3′ part contains the major Pol encapsidation signal (PES) and four purine-rich sequence elements (PPT A-D) [58,59,60]. PPT-D has been demonstrated to function as a second internal or central PPT (cPPT) serving, similarly to what has been reported for HIV, as an additional initiation site for plus-strand DNA synthesis during FV reverse transcription [58,59,61]. PPT-A and -B appear to be essential elements of the PES element of CAS-II and have been attributed functions in promoting FV PR-RT-RH dimerization (protease-activating RNA motif, PARM) and through it also possibly Pol precursor encapsidation [62].

### 2.5. Potential Contribution of Other Foamy Viral and Cellular (co)Factors

Recently, a cluster of high-abundance miRNAs with a unique precursor pri-miRNA has been detected in different SFVs and BFV [63,64,65]. Elimination of the whole miRNA cassette from non-coding sequences in the U3 region of the long terminal repeats (LTRs) does not grossly affect structural protein expression, processing and particle release but it does impair overall BFV fitness and replication competence. These data indicate that the miRNAs do not play an obvious or prominent role in FV assembly and release [66].

FV gene expression and the basic aspects of particle assembly and release including the ESCRT-machinery-directed pinching off of infectious virus seem to be possible in cell lines from different organs and non-authentic, heterologous host species [67,68,69]. This indicates a low level of cell- and species-specific requirements of FVs in this regard. However, as described below, FV particle assembly is restricted to the microtubule organizing center (MTOC) and particle budding is targeted to different degrees to intracellular membranes (e.g., PFV) [70] or to the plasma membrane (e.g., FFV) [71,72]. Additionally, incoming particles are routed to the MTOC highlighting the importance of this sub-cellular site as well as the dependence on the intracellular trafficking machinery of the cell for this bi-directional transport of the FV capsids [73]. These aspects as well as the potential functional importance of these sites and processes are discussed in more detail in Section 3.1 and Section 4.3. 

Finally, FV replication, including the processes of particle assembly and release, is also targeted by host-encoded restriction factors and intrinsic immunity, for instance in the form of APOBEC3 cytidine deaminase packaging, membrane tethering, and interference with full particle release by BST-2/Tetherin and restriction by TRIM5 proteins [74,75,76]. These aspects are not covered here, but the interested reader is referred to a recent review, which does discuss this aspect [4].

## 3. The Mechanisms of Cytosolic Capsid Assembly

FV exhibit a B/D type morphogenesis pattern that follows a two-step process of cytoplasmic capsid assembly and subsequent membrane targeting and budding of the preassembled capsid [3,5]. On the viral side, the following details about these steps in the FV replication cycle that culminate in the release of infectious virions are known:

(i) The identification of the essential viral factors;
(ii) The characterization of their essential functional determinants;
(iii) The designation of the cytoplasmic location of capsid assembly;
(iv) An idea of the subcellular locations of budding across membranes.

At current, the following questions are still mostly unanswered:

(i) The subcellular locations of initial Gag–vgRNA interaction and Pol encapsidation as well as the sequence of these events;
(ii) The sequence of Gag and Pol precursor processing, as well as reverse transcription (RTr) initiation and its possible role in virus maturation during or after capsid assembly;
(iii) The fate and role of the C-terminal p3/4 Gag peptide.

### 3.1. Cytoplasmic Assembly of the FV Capsid

FVs preassemble their capsid in a B/D type dependent manner in the cytoplasm of the infected cell. Like orthoretroviruses, the major protein component of FVs driving capsid assembly is the Gag protein [5]. This is exemplified by the appearance of assembled capsids near centrosomes of cells only expressing FV Gag [70,71,77,78,79]. Several functional motifs/domains within FV Gag have been identified which are essential for capsid assembly at host cell centrosomes (Figure 2a). This includes a cytoplasmic targeting and retention signal (CTRS) with homology to the Mason–Pfizer monkey virus (MPMV) CTRS, which directs assembly to the centrosomal location [77,80]. However, unlike MPMV, FV capsid assembly cannot be simply redirected to the plasma membrane by altering key residues of the CTRS. Introduction of similar aa changes into the PFV CTRS abrogates capsid assembly [77].

Another trafficking signal identified within PFV Gag is a nuclear export signal (NES) [81] (Figure 2a). It is proposed to be responsible for the active nuclear export of Gag after its nuclear interaction with vgRNA, similarly to what has been reported for Rous Sarcoma Virus (RSV) [82]. The authors of this study believe that the temporal nuclear trafficking of Gag, achieved by a chromatin binding signal (CBS; in the GR-rich region of Gag)-mediated import and a subsequent NES-mediated export, is a mechanism for selective encapsidation of vgRNA during FV capsid assembly [81,83,84]. However, this view is challenged by other studies, which either fail to detect any involvement of PFV Gag in nuclear vgRNA export [85], or suggest that Gag nuclear localization is a passive process achieved only by CBS-mediated chromatin tethering upon nuclear membrane breakdown during host cell mitosis and not by active nuclear import of Gag into interphase cell nuclei [86]. Further details about the mechanism of selective vgRNA encapsidation, with the exception of the characterization of putative packaging sequences within the FV RNA genome (see above), are currently not available.

Several Gag motifs essential for correct FV capsid assembly have been characterized. A coiled-coil motif (CC2) located in the N-terminal part of PFV Gag has been shown to be important for Gag-Gag interactions, whereas the neighboring CC3 motif appears to serve as docking site for dynein motor protein complexes enabling trafficking of incoming capsids towards the centrosome during FV entry [73,87] (Figures 2a and 5a). Determination of the crystal structure of an N-terminal, MA-like domain of PFV Gag has revealed that the two CC domains (CC2, CC3) form an extended, single coiled-coil structure essential for dimerization of this domain [25] (Figure 2a(i)).

In addition, mutation of an evolutionary conserved YxxLGL motif located upstream of the GR-rich C-terminal domain of PFV Gag has been reported to result in aberrant capsid assembly and therefore designated assembly (A) motif [88] (Figure 2a). The A-motif is part of a central PFV Gag CA-like domain, whose structure was recently determined by NMR spectroscopy [24] (Figure 2a(ii)). This structure reveals the presence of two all α-helical domains (NtD_CEN_) and (CtD_CEN_) that, although having no sequence similarity, both share the same core fold as the N- (NtD_CA_) and C-terminal domains (CtD_CA_) of archetypal orthoretroviral capsid protein (CA). The tyrosine residue of the A-motif at the C terminus of α-helix 9 in the PFV CtD_CEN_ appears to be involved in hydrophobic interactions forming part of the core of the CtD_CEN_ bundle. In contrast, the LGL portion of the A-motif is exposed and forms a continuous hydrophobic surface patch together with another conserved PGQA motif in α -helix 8. There is speculation that this hydrophobic surface patch is involved in interactions that give rise to hexameric assemblies analogous to those formed in orthoretrovirus capsids [24]. The study of Ball and colleagues [24] has also identified a previously unknown hydrophobic interface between PFV NtD_CEN_ and CtD_CEN_ (Figure 2a(ii)). The importance of this interface for correct capsid assembly and generation of infectious virions has been demonstrated by cryo-electron tomography and infectivity analyses of viral mutants with alterations in key residues of this hydrophobic interface [24].

**Figure A1:**
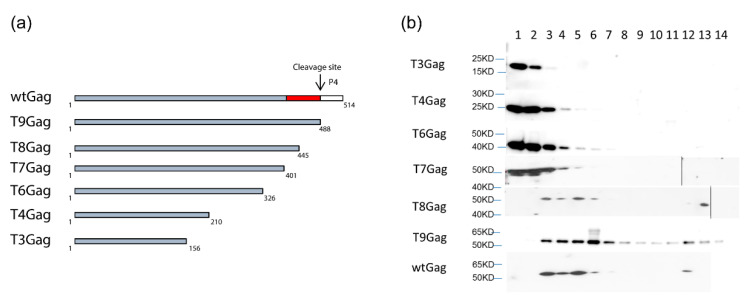
FFV capsomere and capsid formation by wt and C-terminal FFV Gag deletion mutants. (**a**) Schematic of the FFV C-terminal Gag deletion mutants with p4 and the GR-rich domain marked by white and red boxes, respectively. (**b**) HEK293T cells were transfected in 10 cm dishes with 12 μg truncated or wt Gag expressing plasmid. At 36 h post transfection, cytoplasmic extracts were prepared and ultracentrifuged through a 10%–60% continuous sucrose gradient at 32,000 rpm for 2 h. For each sample, 20 fractions (from 1 to 20) were collected from the top of the gradient and analyzed by immunoblotting by using an anti-Gag MA antiserum. Fractions 15–20 are not shown because no bands were visible. Fractions 1–2: unassembled Gag proteins; fractions 3–7: capsomeres; fractions 12–14 capsids. Shortened from detailed data in [92].

Neither Pol nor vgRNA are essential for FV capsid assembly since the expression of Gag alone is sufficient for particle formation [89,90]. However, Pol encapsidation and PR-mediated processing of the Gag precursor result in a larger fraction of completely closed capsid structures to be assembled at the centrosome (see below). Capsids formed by the PFV Gag precursor alone, in absence of Pol coexpression, or in presence of PR-inactive Pol variants, display a high frequency of incompletely closed capsid structures of horseshoe like morphology [90]. It can be assumed that interaction of Gag with nucleic acids in general, but not necessarily with vgRNA, through its C-terminal GR-rich domain is a prerequisite for the assembly of FV capsids with normal morphology or capsid-like structures. C-terminal PFV and FFV Gag truncation mutants as well as full-length PFV Gag with 23 arginine residues of the C-terminal GR-rich domain changed to alanine are unable to assemble normal shaped capsids. This is most probably due to their inability to interact with cellular or viral nucleic acids [91,92] (Figure A1). Thus, Gag-nucleic acid interactions are fundamental for FV capsid assembly similar as for all other retroviral Gag proteins.

We can speculate that the binding of Gag to currently unknown cellular RNAs via the C-terminal GR-rich domain drives Gag multimerization into capsomeres that are the true building blocks of particle assembly. This theory is based on sedimentation analyses of cytosolic extracts containing wt and a family of closely-spaced C-terminally truncated FFV Gag proteins [92] (Figure A1a). Full-length, wt, and some C-terminal truncations yield high molecular mass assemblies corresponding to capsids and lower order assemblies with a currently undefined degree of oligomerization (Figure A1b, fractions 11–14 and 3–7). Following the deletion of almost the entire GR-rich domain, such lower order assemblies and capsid-like forms are no longer detectable. These truncated Gag proteins remain on top of the gradient. Putative capsomeres (Figure A1b, fractions 3–7) may form at the MTOC or at the site of translation before retrograde transport to the MTOC. The latter scenario may fit to the observation that all wt and assembly-competent FFV Gag enter the gradients as higher order assemblies and would, additionally, reflect the economy of cellular transport systems and the utilization of preformed building blocks in virus assembly [5,93].

### 3.2. Unique Pol Packaging Concomitant to Genome Encapsidation

As mentioned above, FVs are unique amongst RVs as they translate Pol as a separate protein from a spliced subgenomic mRNA rather than from the full-length genomic RNA as Gag-Pol fusion protein [29,30,33]. This feature necessitates a Pol encapsidation strategy that deviates from the standard orthoretroviral mechanism that ensures enzyme packaging into the assembling capsid through Gag-Gag interaction of Gag and Gag-Pol precursor proteins. It is generally accepted that FV Pol encapsidation requires Pol interactions with vgRNA via specific CAS elements containing PES (Figure 3) as well as binding of other CAS elements within the vgRNA by the GR-rich C terminus of Gag (see Section 2.4). Under natural Pol expression conditions within the proviral context, only the Pol precursor (PFV pr127^Pol^), but not the mature p85^PR-RT-RH^ and p40^IN^ subunits (Figure 2b), are encapsidated into assembling FV capsids in a strictly vgRNA-dependent fashion [55,60]. Under conditions of cellular Pol overexpression in trans, significant cellular amounts of mature PR-RT-RH and IN subunits are detectable, and the Pol precursor as well as mature subunits can be packaged into assembling FV virions, in a vgRNA-independent manner [22]. Therefore, FV vgRNA appears to serve as a scaffold for assembly of Gag and Pol into infectious viral capsid structures. In contrast, the contribution of direct Gag-Pol protein-protein interactions for Pol packaging are discussed controversial [35,94]. In addition, overexpression of Pol results in Gag- and vgRNA-independent cytoplasmic Pol processing [30].

Details of the temporal and spatial regulation of FV vgRNA and Pol encapsidation have not been characterized. It is therefore unclear whether all three components, vgRNA, Gag, and Pol are transported separately to the centrosome and capsid assembly initiates and proceeds at this key organelle in the FV replication cycle or if assembly intermediates encompassing two or all components are formed before or during transport to the centrosome. The order of protein–nucleic acid interactions is currently also unknown. Does Gag or Pol first interact with vgRNA or do both bind simultaneously, perhaps enhanced by additional protein-protein interactions?

## 4. The Mechanisms of Membrane Acquisition and Budding

### 4.1. Unique Particle-Associated N-terminal Stable FV Elp/LP 

**Figure 4:**
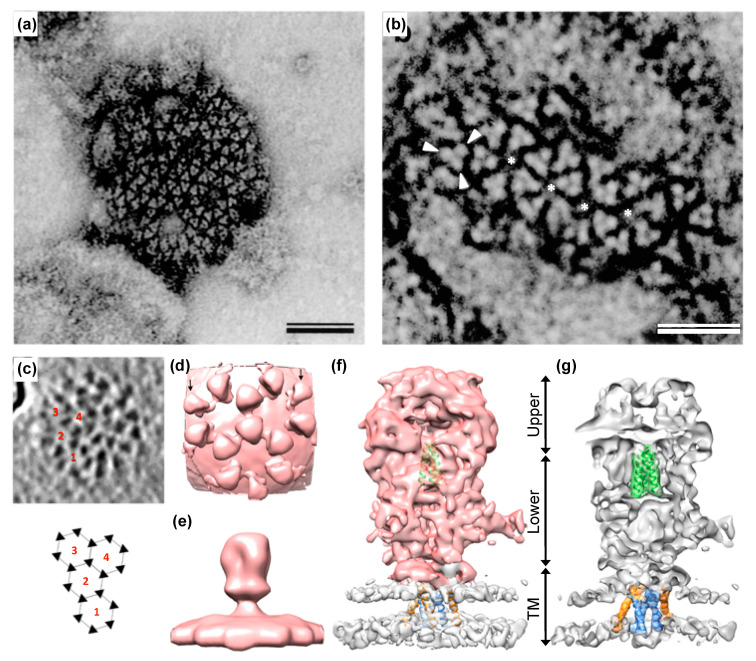
Structure of FV envelope glycoprotein complex (GPC) and the GPC lattice of FV virions. (**a**) Surface features of PFV particles detected by negative-staining EM. Virions show a network of trimeric viral spike proteins on the particle surface and are predominantly arranged into rings of six subunits. Adjacent rings always share two completely integrated spikes. Images at higher magnification (**b**) reveal three separate densities (arrowheads) in the triangular spike. When grouped in hexameric rings, a stain-filled hole with a diameter of about 8 nm was formed (asterisk). Bars represent 50 nm (**a**) and 25 nm (**b**); (**c**–**e**) subtomogram averaging of PFV glycoprotein. (**c**) 0.8 nm thick tomographic slice perpendicular to the glycoprotein long axis and its corresponding schematic of interlocked hexagonal assemblies of trimers. Numbers are indicated at the center of each hexagon and triangles represent the position of each trimer of Env in the hexagonal network. (**d**) Top view of intertwined hexagonal assemblies. (**e**) Side view of a single trimer. (**f**–**g**) In Situ single particle 3D reconstruction of PFV glycoprotein by cryo-EM. Full (**f**) and cut-away (**g**) side views of a single PFV Env trimer (sharpened map) after 3-fold symmetry application (~9 A resolution at FSC = 0.143). The densities corresponding to the extracellular domains and the viral membrane are colored salmon and gray respectively in (**f**). The three central helices attributed to gp48 fusion peptide are represented by three green α helices of 22 residues long each. The transmembrane helices (TMHs) are represented by three inner (colored blue) and three outer (colored orange) α helices. In (**g**), the densities surrounding the three central helices and the three inner and outer TMHs are colored green, blue and orange respectively while the remaining of the spike is gray colored. Panel (**a**,**b**) adapted from [95]; panel (**c**–**g**) adapted from [7].

As mentioned above, the mature tripartite and trimeric Env GPC found on released PFV and FFV particles (Figure 1) is unique amongst RVs as it includes three separate subunits, Elp/LP, SU, and TM, all derived by posttranslational proteolytic processing from the same Env precursor [6,41]. High resolution or crystal structures of the mature FV Env GPC are currently not available. Though, some structural information at lower resolution was obtained by image reconstruction analysis of negative stain electron micrographs or cryo-electron tomographs and -micrographs [7,95] (Figure 4). For example, these data reveal that the full particle surface is covered by a dense and highly repetitive arrangement of the prominent FV GPCs. They are placed in an elaborate lattice on the surface of virions consisting of overlapping hexameric rings of Env trimers, which are easily distinguishable using negative stain electron microscopy [6,7,95] (Figure 4a–c). The high order and dense arrangement of adjacent GPC trimers strongly suggest that lateral interactions between the trimers are a prominent feature of the FV surface protein assemblies (Figure 1d and Figure 4a–d). In fact, the lateral interaction may provide the energy for particle budding. This may explain the Env-dependence of FV budding and the ability of FV Env proteins to induce release of capsid-less SVPs harboring only the GPC [6,7,52,95,96]. The structures of individual FV GPCs at ~10 Å resolution were obtained using cryo-electron tomography or -microscopy analysis of PFV particles with different glycoprotein variants [7] (Figure 4e–g). They support a heterotrimeric GPC organization and strongly suggest that the Elp/LP subunit is an integral component of the FV GPC together with the SU and TM subunits (Figure 1a).

### 4.2. Unique FV Elp/LP-Dependent Envelopment and Release 

The unique requirement for co-expression of Env in addition to Gag for release of FV VLPs had been recognized early on [89,90,97]. Unraveling the major principles of FV capsid membrane association, lipid membrane envelopment, and particle egress as well as the characterization of the Gag and Env determinants involved in PFV and FFV has required considerable time, and there are still several gaps of knowledge that need to be filled. The discovery of the unique biosynthesis and posttranslational processing of the FV glycoprotein in PFV and FFV [6,39,40,41,42] has been groundbreaking for the mechanistic understanding of these processes. With an Env precursor having CyDs at the N and C terminus, it has become obvious and probable that interactions of the C-terminal CyD of the TM subunit with the capsid, as observed for other RVs are not the only ones involved in FV particle release. The FV capsid/Gag also interacts with the Elp/LP N-terminal CyD subunit. It has been found that a region approximately comprising the 15 N-terminal aa of LP, harboring two conserved and closely and invariantly spaced tryptophan residues (WxxW), and a coiled-coil domain (CC1) in the N terminus of FV Gag are the major essential determinants for FV budding and particle egress (Figure 2a,c) [6,25,41,98]. The MSD of the FV TM subunit appears to contribute to budding to some extent as well, but exactly how is currently unclear [97]. The early studies using surface plasmon resonance (SPR) and co-immunoprecipitation (co-IP) analysis to define an N-terminal Elp/LP “budding domain” suggested and demonstrated that a direct interaction of Elp/LP and Gag with both tryptophan residues is critical and essential [6,41,98]. Indeed, this has been confirmed by solving the crystal structure of an N-terminal MA-like domain of PFV Gag comprising aa 1-179 and variants thereof co-crystallized, with peptides representing the N terminus of PFV Elp/LP (aa 1-20) (Figure 2a, i) [25]. These studies demonstrate a direct interaction of both domains, with the two conserved Elp/LP tryptophan residues contributing the major anchoring hydrophobic interactions.

### 4.3. Transfer of the Fully Assembled Capsid to the Site(s) of Particle Envelopment and Budding

Like the B-type RVs MPMV and MMTV with a MTOC-targeted B/D-type capsid assembly and packaging strategy, FVs face the need to transport the newly assembled capsid to the site of envelopment and budding at different cellular membranes [99]. There is good evidence that MPMV uses microtubule- and kinesin-mediated anterograde transport of Gag/capsids and Env to the budding site(s) [100]. While currently unknown, it seems likely that FVs also use anterograde microtubule-mediated transport to target full capsids to the sites of release, either the intracellular membranes of the Golgi network or the plasma membrane [70]. In the light of the data on MPMV Gag/capsid-Env co-transport from the MTOC to the cell periphery [100], it is possible (though this is speculation) that the first physical interaction between the FV Elp/LP and Gag/capsids occurs at the MTOC assembly site. In this scenario, FV Env-containing transport vesicles may target the MTOC with assembled capsids allowing the interaction of the N termini of the MA-domain of assembled capsids and those of Elp/LP in the transport vesicles. While the microtubule-mediated transport of diverse cargo vesicles is a well-established cellular process [93], the interaction of corresponding Env-containing vesicles with FV capsids has, to our knowledge, not yet been described or detected.

The signals targeting FV Gag and capsids to the MTOC or being required for microtubular transport, are located upstream of the CA-domain in the MA-domain or MA-layer/intermediate shell [73,77]. Since they are required for both, antero and retrograde transport, their proteolytic separation from the capsid-forming domain would interfere with the early retrograde transport of the incoming capsid towards the MTOC/nucleus. This functional link in FVs may be one of the reasons for this lack of conventional processing in an orthoretroviral fashion.

Currently, the kinetics of the retrograde transport of MTOC-associated capsids to the site of particle release are completely unknown but their accumulation and the ease of detecting them by electron microscopy argues for low- or moderate-speed transport [70,71,78,79]. This would allow the influx of dNTPs into the full but non-enveloped particles in the cytoplasm over an extended period of time. This is not comparable to the situation in those RVs where particle assembly, envelopment, and budding co-occur. Since all structural and mechanistic studies on FVs have been done in immortalized, transformed cells, one can assume that dNTP levels within these tumor cells are unphysiologically high, thus providing suited concentrations to allow cDNA synthesis already in the virus-producing cell. However, the same applies to other retroviruses utilizing a B/D type assembly strategy, such as MMTV or MPMV, but they do not initiate RTr in preassembled capsids. Therefore, as discussed (see Section 2.2 and Section 4.5), the relaxed control of PR and RT activity of the FV Pol precursor, which is not observed for other retroviruses, may be primarily responsible for the (partial) reverse transcription of the FV RNA genome already in the virus-producing cell. It would be highly interesting to examine the level of FV reverse transcription in primary cells. On the other hand, if a tripartite complex consisting of the FV Pol precursor, vgRNA and Gag/capsomeres is preformed in the cytoplasm before intracellular transport (see above, Section 3.2.) or before capsid assembly is completed at the MTOC, reverse transcription may already be initiated at this point and may be even required to stabilize such assembly intermediates. Additionally, impairments in innate immunity, including the sensing of cytoplasmic viral nucleic acids or other viral components, is often a characteristic of transformed cells—a feature that may increase cell permissiveness towards DNA synthesis within the virus-producing cell/cytoplasm.

In summary, the importance of the MTOC and the microtubular network for FVs and B-type RVs as a central hub in their replication is underlined by the fact that free Gag or assembly intermediates—Gag proteins or higher order assemblies thereof (capsomeres)—as well as incoming virus particles (and/or the pre-integration complex), are concentrated at the MTOC. In these cases, dynein or kinesin (in B-type RVs) have been shown to mediate this microtubular transport.

### 4.4. Envelopment and Release

Unfortunately, only few data about the influence of Env GPC maturation (precursor processing, oligomerization, posttranslational modification) on FV egress are available. Whereas inactivation of the SU/TM furin cleavage site has no influence on FV release but abolishes viral infectivity, inactivation of the Elp/LP–SU furin cleavage site, or introduction of mutations in its neighborhood result in a strong reduction of FV particle release, but have only minor effects on their specific infectivity [39,40,101,102].

It is unclear at which stage of FV Env GPC maturation the interaction with assembled capsids takes place while it travels through the cell’s secretory pathway. Although purely speculative, we can envision a scenario of preassembled capsids interacting with the cytoplasmic N terminus of the Elp/LP domain in the context of the full-length Env precursor embedded in transport vesicles of the secretory pathway. This interaction may result in efficient co-transport of capsid–Env complexes and/or induction of budding structures at cellular membranes. Perhaps budding is initiated or enhanced by furin-mediated proteolytic processing of the Env precursor and may subsequently alter the affinity of the Gag–Elp/LP interactions. It may be important for FVs to enable a transient Gag–Env interaction that allows release of the capsid into the cytoplasm after membrane fusion during target cell entry due to the limited processing of FV Gag that does not yield a separate MA subunit as found in other RV particles. Strikingly, cryo-electron tomography analyses of FFV virions during and after budding point to differences in the distance of putative MA layers to lipid membranes (Figure 1). In budding intermediates, the MA layer is closely spaced and exactly follows the curvature of the membrane whereas the MA layer in released particles follows the curvature of the capsid and is only at a few sides close to the viral membrane [6]. 

A consequence of the strict Env-dependency of FV budding and particle egress is the inability to naturally pseudotype FV capsids with heterologous viral glycoproteins [97]. This is mainly due to the absence of membrane-targeting or membrane-association signals (MTS) in FV Gag proteins [89,90]. Only N-terminal addition of heterologous MTS sequences enables the release of FV VLPs similar to what is a natural feature of orthoretroviral Gag proteins [77,78,103,104,105,106]. However, genetic fusion of MTS to the N terminus of Gag results in VLPs that are non-infectious, even if all other components required for assembly of an infectious virion (Pol, Env, vgRNA) are co-expressed and the natural FV Gag–Elp/LP interaction is inactivated [77,78,103,104,105]. This is probably due to an aberrant capsid assembly during virus morphogenesis and a potentially impaired capsid disassembly upon target cell infection as a consequence of the limited proteolytic processing of FV Gag proteins [78,104]. Unlike their orthoretroviral analogs, the FV Gag proteins with N-terminal MTS may be permanently membrane-associated. Orthoretroviral Gag proteins only show a transient membrane association of the Gag precursor during budding and a subsequent membrane release of the CA and NC subunits by proteolytic separation from the membrane targeting MA-domain during virus maturation [5]. A transient membrane association of FV Gags appears to be essential for FV replication too, since reversible linkage of heterologous MTS to PFV Gag using a small-molecule regulated protein–protein heterodimerization system enables the production of fully infectious FV virions in a FV-Env independent manner, even with heterologous viral glycoproteins [105]. We believe that the membrane interaction of FV Gag is regulated by the highly specific FV Elp/LP–Gag protein–protein interaction, which presumably is also transient in nature as indicated also by electron microscopy of budding intermediates and released FFV particles [6].

### 4.5. Absence of Release-Dependent Canonical Gag Processing 

As pointed out earlier, one hallmark of FV morphogenesis is the very limited Gag proteolytic processing that is mostly or exclusively restricted to a single site in the Gag precursor (PFV pr71^Gag^) (Figure 2a). This leads to the formation of capsids of apparent immature morphology in released PFV virions, which are composed of unprocessed Gag precursor and the large cleavage product (PFV p68^Gag^), at variant /variable 1:1 to 1:4 ratios in all FVs studied so far [107].

We know that FV particles composed of only the Gag precursor (pr71^Gag^ only), obtained either by inactivating the Gag cleavage site or the catalytic center of the Pol PR domain, are non-infectious even if they otherwise contain all other essential viral structural components (Pol, vgRNA, Env), because they completely fail to reverse transcribe their packaged vRNA genome [22]. This type of FV virion shows a higher frequency of irregularly shaped, not completely closed capsids. From the characterization of other FV Gag mutants displaying aberrant capsid morphologies it appears that a correct capsid assembly and shape are prerequisites for RTr, either intraparticle during or after assembly, or during and subsequent to target cell entry [22,24,88].

FV particles composed of only the large Gag processing product (p68^Gag^ only) and lacking the Gag precursor on the other hand display a capsid indistinguishable from wild type [90]. Furthermore, they are still infectious, although their specific infectivity is 10- to 50-fold reduced compared to wild type [22]. This appears to be the consequence of a reduced RTr efficiency since the reduction in their specific infectivity correlates with a similarly reduced frequency of intraparticle genomic reverse transcripts. Thus, for optimal RTr efficiency, a defined ratio of pr71^Gag^ and p68^Gag^ appears to be essential. How this can be mechanistically explained is currently unclear.

**Figure 5:**
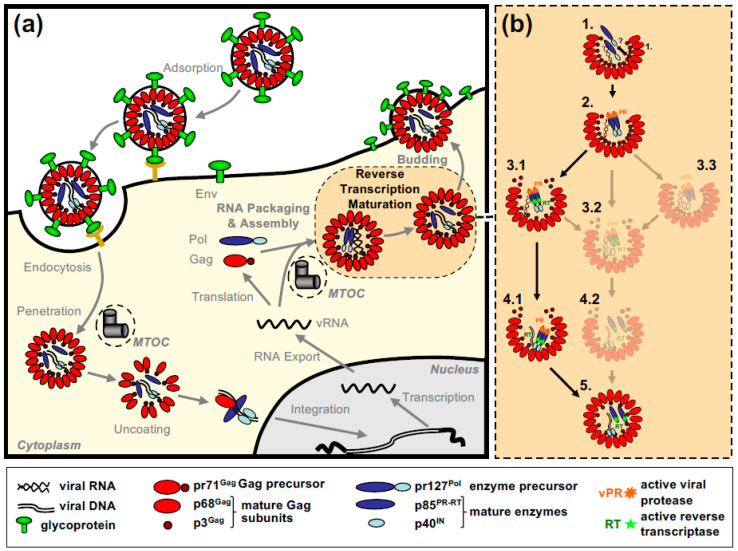
Schematic illustration of FV replication and virion maturation. (**a**) Simplified schematic illustration of the FV replication cycle. FVs attach to target cells by interaction with cell surface heparan sulfate. Virions having already undergone RTr during assembly and release (up to 20% of total) contribute to the majority of productive infection events. Virions enter the cell by endocytosis and capsids are released into the cytoplasm by a pH-regulated fusion of viral and cellular lipid membranes mediated by FV Env and unknown cellular entry receptor(s). Intact capsids migrate along microtubules to the centrosome where they remain in a latent state until the host cell enters mitosis. This induces further disassembly and enables chromatin access of the FV pre-integration complex upon nuclear membrane breakdown. Following integration and FV Tas-mediated transcription regulation viral RNAs are transported to the cytoplasm or the ER where protein translation takes place. Further details of the late steps of FV replication are discussed and summarized throughout the manuscript. (**b**) Model of sequential events resulting in infectious FV capsid maturation. 1. FV Pol is incorporated predominantly at the precursor state involving binding of Gag (or capsomeres) and Pol to vRNA and potentially additional Gag–Pol protein interactions; 2. Binding of Pol to the PARM elements of the vgRNA and potentially oligomerization by the IN domain result in PR domain dimerization associated with PR activation; 3. Different possibilities exist for subsequent Gag and Pol precursor maturation. First either Gag (3.1) or Pol (3.3) is cleaved followed by the cleaving of the other component, or both proteins (3.2) are cleaved simultaneously. 4. Gag precursor processing activates RT activity either in the Pol precursor (4.1) or the mature PR-RT subunit (4.2); 5. This leads to RTr of the packaged vRNA genome in up to 20% of all released particles, which contribute to the great majority of FV infectivity. The processing pathways that are less likely in the opinion of the authors are indicated by lighter representation.

The temporal and spatial regulation of Gag and Pol precursor processing by the viral PR domain of Pol during capsid assembly is in large parts a black box in FV morphogenesis. We neither know the location of Gag processing during its intracellular transport nor its timing during capsid assembly and the sequential order of Pol and Gag precursor cleavage during this process (Figure 5b). It appears that both Pol precursor and the mature PR-RT-RH subunit harbor enzymatically active PR and RT domains and that Pol can be processed at the IN cleavage site independent of Gag/capsids [22,30]. FV PR requires dimerization to become enzymatically active. This is thought to be achieved by either binding to the PARM element in CAS-II of the vgRNA and/or protein–protein interactions of the IN domain in the Pol precursor [62,94,108]. However, to our knowledge a direct comparison of the relative PR and RT activities of precursor versus mature subunit has not yet been performed.

Unlike orthoretroviruses, which initiate final viral maturation by Gag and Gag-Pol precursor processing only during or shortly after viral budding, FV capsids assembled at the centrosome already contain processed Gag and Pol proteins prior to their membrane envelopment and cellular egress. The failure to detect the small C-terminal Gag processing product p3^Gag^ in released PFV virions may indicate that Gag precursor processing starts before the capsid is fully assembled and this small processing product thereby evades encapsidation (Figure 5b). Alternatively, this may be simply explained by a lack of sensitivity of classical methods, such as Western blot analysis, employed so far for this analysis, as a consequence of the low molecular weight of p3^Gag^. Unfortunately, a proteome of highly purified FV particles, which may provide more information about the presence of absence of p3^Gag^ in released FV virions, has not yet been determined.

In vitro, RTr is initiated during virus morphogenesis resulting in the release of a mixture of vDNA or vRNA containing virions in 5 to 20% of all assembled capsids [32,109,110]. In our study, the initiating event for RTr is Gag precursor cleavage that results in generation of the PFV p68^Gag^ processing product [22], although another study [108] suggests that this event is regulating the template switch during RTr (Figure 5b).

## 5. Interconnection and Co-Evolution of Unique or Non-Canonical Mechanisms

### 5.1. FVs as the Most Ancient Vertebrate RVs: Conservation of Archaic Retroviral/Retroid Traits?

Few distinct genomes of EndFVs have been detected in all classes of the vertebrate tree from coelacanth through to mammals and evolutionary analyses suggest that FVs are the most ancient (vertebrate) RVs [9,10,11,12,13]. The finding that FVs are at the roots of the retroviral tree may indicate that what is now described and discussed here as deviant features of the *Spumaretrovirinae* when compared to the *Orthoretrovirinae* may be, at least partly, archetypical traits of the RV family. In contrast, the more-recent orthoretroviruses may be characterized by defined newly acquired evolved and advanced molecular mechanisms of virus replication. We will develop this idea further with respect to the “unique but archetypical” strategies of today exogenous FV particle assembly, release and maturation.

While structural details have been resolved for all parts and mature cleavage products of orthoretroviral Gag proteins, this is not the case for FV where only the structures of the N terminus corresponding to the MA-domain and the central CA-domain are available for PFV (Figure 2a). However, these structures are likely to be representative for the whole FV subfamily [24,25]. The FV central Gag domain is clearly structurally related to the orthoretroviral CA proteins [24] and to the folding of CA in mature LTR retrotransposon (*Metaviridae*) capsids [111]. In contrast, no obvious similarity has been described for the MA proteins/domains of spuma- versus orthoretroviruses [25]. Finally, according to bioinformatics analyses using Gag sequences of RVs and diverse retroid elements, the NC-domain of FVs has no counterpart among these elements [112]. Thus, the overall image of the central FV Gag CA-domain shows conserved retroviral/ortervirales traits, while this is not the case for the N-terminal MA- and the C-terminal NC-domain, and that their evolutionary origins are not known at current.

**Figure 6:**
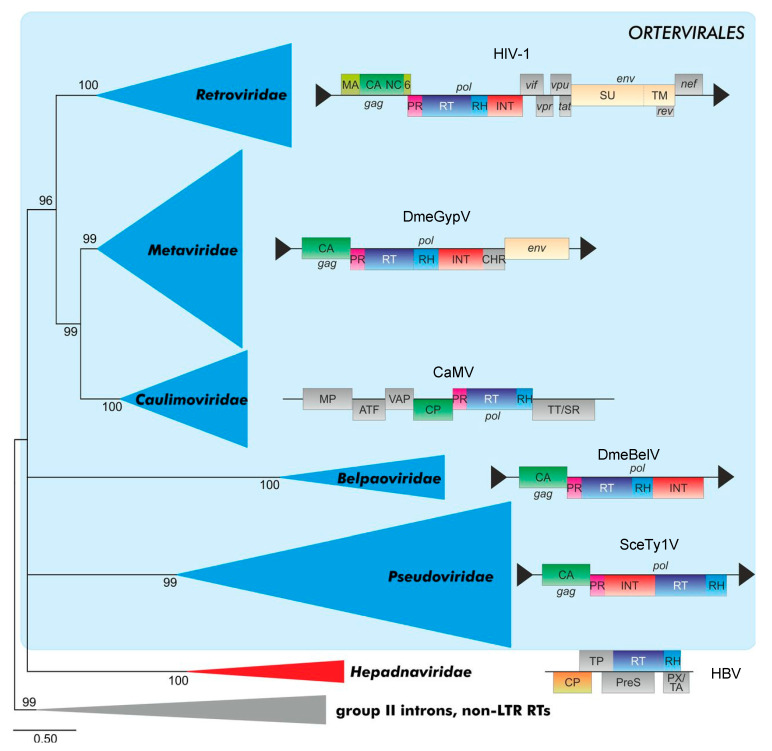
Maximum-likelihood phylogeny of viral reverse transcriptases. The tree includes sequences of 290 viruses belonging to all International Committee on Taxonomy of Viruses (ICTV)-recognized genera of reverse-transcribing viruses. The phylogeny was inferred using PhyML [115] with the LG + G + F substitution model and is rooted with sequences from nonviral retroelements (bacterial group II introns and eukaryotic LINE retroelements). Genomic organizations of selected representatives of reverse-transcribing viruses are shown next to the corresponding branches. Long terminal repeats are shown as black triangles. Note that members of the virus families display considerable variation in gene/domain content [116], which is not captured in this figure. Abbreviations: 6, 6-kDa protein; ATF, aphid transmission factor; CA/CP, capsid protein; CHR, chromodomain (present only in the integrase of particular clades of metaviruses of plants, fungi, and several vertebrates); gag, group-specific antigen; env, envelope genes; INT, integrase; LTR, long terminal repeat; MA, matrix protein; MP, movement protein; NC, nucleocapsid; nef, tat, rev, vif, vpr, and vpu, genes that express regulatory proteins via spliced mRNAs; P, polymerase; pol, polymerase gene; PR, protease; PreS, pre-surface protein (envelope); PX/TA, protein X/transcription activator; RH, RNase H; RT, reverse transcriptase; SU, surface glycoprotein; TM, transmembrane glycoprotein; TP, terminal protein domain; TT/SR, translation trans-activator/suppressor of RNA interference; VAP, virion-associated protein. HIV-1: *Human immunodeficiency virus 1*; DmeGypV: *Drosophila melanogaster gypsy virus*; CaMV: *Cauliflower mosaic virus*; DmeBelV: *Drosophila melanogaster Bel virus*; SceTy1V: *Saccharomyces cerevisiae Ty1 virus*; HBV: *Hepatitis B virus*. Image and caption adapted from [2].

The functionally and structurally conserved capsid-forming CA moiety of an ancestral retroid/retroviral Gag precursor may have served as the crystallization or starting point for the evolution to the present day RV Gag proteins with their high level of diversity between and within both retroviral subfamilies. We propose the subsequent acquisition of one or two domains with additional functions flanking CA in the diverse branches of the *Ortervirales* (Figure 6). Two different module “types” allowing specific interaction with RNA, DNA and DNA/RNA hybrids were added to the C terminus or evolved de novo, one of the Cys-His finger type whereas the other represented the GR-rich basic histone-like type present in Caulimo- and Spumaviruses. The basic histone-like elements of the only distantly related hepadnaviruses may represent a convergent development [2]. The retroid elements that acquired an additional *env* gene gained at the N terminus of the CA-domain a module responsible for membrane targeting and/or Env interaction to allow capsid envelopment and release ([113,114] see below, Section 5.2).

For FV Env, most structural details are also unknown but the overall topology and presence of key elements of the SU- and TM-domains (the SU–TM processing site, the hydrophobic transmembrane and fusion domains of TM and the short cytoplasmic tail of TM) appear to be similar to most other RVs [37,117]. However, as discussed above, the N terminus of FV Env is fundamentally different, which is reflected by the large size of Elp/LP-domain (120 to 130 aa residues), its unconventional processing by furin proteases, and its high stability and abundance in released virus particles. The combination of these features is unique among RVs and their relatives. In orthoretroviruses, the N terminus of Env is usually much smaller (up to about 35 aa residues) and mostly required for ER targeted expression and translocation into the ER lumen. Exceptionally large SPs with a considerable stability have been described for MMTV [44,45] and RSV [118]. The MMTV Env SP comprises 98 residues, harbors a nucleolar localization signal and functions during export of intron-containing transcripts [44,45]. The N terminus of the 36 aa RSV Env SP is encoded by the *gag* ORF and added to the *env* ORF via splicing [118]. However, the above average size of the Env leader sequences in MMTV and RSV appears to be unrelated to any function during particle assembly and release in these viruses and may represent the acquisition of additional functions/traits. In contrast, and as mentioned above, FV Elp/LP is essential for the envelopment and release of particles [6,41].

We consider it likely that at least some of these FV-specific and possibly ancient traits in Gag and Env, the basic retroviral assembly and release machinery, are functionally linked by a long co-evolutionary history of both proteins. However, the unique Pol protein expression strategy, as well as its FV-specific packaging and regulation have likely also contributed to the singularities and complexities of FV assembly, release, and maturation, strongly suggesting a basically different underlying evolutionary history than for the other RVs as discussed below.

### 5.2. Concepts for the Evolution and Co-Evolution of the FV Gag–Env Assembly and Release Machinery

A novel taxonomy of retroid elements proposed by the International Committee on Taxonomy of Viruses (ICTV) in 2018, and based on underlying evolutionary relationships, created a new order of retroid elements, the *Ortervirales* [2]. The *Ortervirales* cover the following five families of reverse-transcribing (retroid) elements carrying in their DNA form (in most cases) LTRs: the *Retroviridae* (*Ortho-* and *Spumaretrovirinae*), the *Metaviridae* (e.g., the Ty3/Gypsy LTR-retrotransposons), the *Pseudoviridae* (e.g., the Ty1/Codia LTR-retrotransposons), the *Caulimoviridae* (e.g., cauliflower mosaic virus of plants) and the *Belpaoviridae* (Bel/Pao retrotransposons both without and with LTR-like terminal sequences). *Hepadnaviridae* were not included in this order since they lack most of the other unifying features of the new order of *Ortervirales*, although they also replicate via a reverse transcriptase like all *Ortervirales*, [2]. The new classification, which is based on evolutionary considerations will hopefully end unnecessary discussions of whether FVs are “the missing link” between the orthoretroviruses and the hepadnaviruses including hepatitis B virus HBV. In the following discussion, we will use the new nomenclature in brackets since most of the literature covering the evolutionary relations of RVs within the *Ortervirales* does not use this new nomenclature of the different groups of retroid elements.

RVs are considered to have evolved from precursor LTR-retrotransposons (*Metaviridae*), in particular Ty3/Gypsy elements, although other evolutionary scenarios have been discussed as well [119]. Within the *Metaviridae* group of LTR-retrotransposons, many members lack a cell-free, infectious phase due to the absence of the retroviral *env* gene (cluster) encoding the cell targeting, binding and entry machinery ([2]; ICTV homepage at https://talk.ictvonline.org/; Figure 6). However, other members of the *Metaviridae* family encode Env-like sequences that (may) facilitate cell-to-cell and even host-to-host transmission events. It is assumed that RVs branched off the *Metaviridae* group of LTR retrotransposons more than once [112].

The Gag (or ORF 1) of Env-deficient LTR retrotransposons only consists of CA- and NC-domains, some even lack NC. It is very likely that an Env-interaction function/domain of Gag is needed for membrane targeting, envelopment, and release in Env-encoding *Ortervirales*. However, in some of the ancient retroid elements or RVs, a membrane-associated particle assembly and/or replication may already have evolved before or concomitantly with the acquisition of an Env-mediated extracellular, infective life cycle. Targeting complex biochemical or structural processes to membranes is a means to increase local concentrations of enzymatic active components or assembly intermediates and serve as a scaffolding mechanism—this is for instance true for several non-enveloped viruses where at least envelopment is not needed [120].

RVs have probably acquired Env more than once [113]. Non-vertebrate LTR retrotransposons may either possess or “lack” an *env* gene and, thus, an extracellular phase. Ancient Gag CA molecules of Ty3/Gypsy LTR-retrotransposons (*Metaviridae*) may have had different flanking N-terminal sequences that allowed interactions with either N- or C-terminal sequences of Env-like membrane proteins. Co-evolutionary processes in such new combinations of Gag- and Env-like molecules of given new RV precursors likely selected then for the most suited partner combinations. This would have finally given rise to at least two distinct groups characterized by a spuma- and an orthoretrovirus-type Gag–Env envelopment and budding machinery. For instance, the unique structure of the current FV MA-domain [25] may be the consequence of the fact that it co-evolved with a FV-type Env protein with stable/strong N-terminal budding and release functions provided by the unique Elp/LP-domain. In contrast, the ancestors of the orthoretroviruses may have gained or preserved two fundamentally different strategies of capsid/particle assembly and release by combining different Gag and Env precursors. One option is a cytosolic capsid assembly and subsequent myristic acid/acetylation-dependent membrane targeting and envelopment as exemplified by (B- and D-type RVs). Alternatively, a membrane-directed or membrane-assisted capsid assembly is followed by the release of Env-studded or Env-free particles (e.g., lentiviruses and C-type RVs). Both orthoretroviral mechanisms result in a mostly Gag-driven and Env-independent particle release mechanism.

Since capsid assembly of Env-deficient retroid elements is mainly, if not exclusively, cytoplasmic, the membrane assisted capsid formation of C-type and lentiviruses may represent a “comparably new” strategy enforced by the newly acquired Env- and budding-mediated extracellular phase. In contrast, RVs with a cytosolic assembly need specific mechanisms to transport the bulky capsid to the membranes for envelopment and release. Most probably, B-/D-type orthoretroviruses and FVs have gained access to cellular transport systems to achieve this task by the acquisition and utilization of adapter motifs as discussed above.

In FVs, under conditions of high-particle density infections, incoming particles are targeted to the MTOC [73], the site where capsid assembly has also been shown to occur. The MTOC is also the site of MPMV and MMTV particle assembly [100]. To our knowledge, their incoming particles are not targeted to this subcellular compartment, which is close to the nucleus. MTOC targeting of MPMV and FV Gag during capsid assembly is considered to be achieved by the CTRS described above. Whether the CC3 coiled-coil domain in the FV MA-domain alone or in combination with the N terminus proximal Gag CTRS is responsible for targeting incoming FV capsids to the MTOC is unclear (see above). Both motifs are present in the PFV MA-domain as defined by structural analyses [24,25]. If either one or both signals are required for MTOC targeting of incoming particles, the proteolytic release of the MA-domain from the capsid would likely interfere with this CTRS and/or CC3-mediated MTOC transport pathway. This idea may provide the reason why FV Gag processing at the N terminus is absent or at least temporarily delayed [20].

In order to address these functional and evolutionary questions in full detail, one has to take advantage of the constantly increasing wealth of sequencing data from different organisms leading to the identification of a growing number and diversity of retroid elements. Here, a combination of advanced tools and methods in molecular evolution and retroviral “paleontology” of endogenous sequences and functional molecular biology/virology concepts and studies may help us gain new insights into ancient viral evolution [121,122].

### 5.3. Spumavirus Assembly: Ancient Mechanisms but Functional and Evolutionary Flexibility

FVs are characterized by a high degree of sequence conservation within a virus species but also between the different genera. Even following host species switches, genomes are highly stable similar to the situation of human and simian T cell leukemia viruses (HTLVs and STLVs) [123]. Similarly to HTLV, the BFV is highly cell-associated, a trait that reduces genetic diversity via repeated, numerous rounds of transmission and reverse transcription events as in HIV with its high level genetic variability within and between infected individuals [123]. However, selection of BFV for a cell-free and high-titer transmission phenotype reproducibly and independently results in the in vitro selection of BFV variants with this novel phenotype within a comparably short time [106,124]. Gag and Env proteins show the presence of adaptive mutations: a comparably small number of such mutations characterize virus populations with high-titer cell free transmission and some individual changes in fact increase transmission [124,125]. Besides evenly spread changes throughout Gag and Env, some mutations co-occur [125]. Surprisingly, the proline-rich part between the Gag MA- and CA-domains even contains substantial deletions and insertions reflecting a high degree of variability especially in this region [124,125]. Thus, budding efficacy in the ancient and highly co-evolved FVs shows a surprisingly high degree of flexibility and adaptability as shown in these in vitro selection and evolution screens. This is in line with other features of FV assembly and release, where genera-specific features are detectable, for instance concerning the ubiquitination of Elp/LP, the size of the MA-layer/intermediate shell, the preferred site of particle budding, and the Gag nuclear targeting/chromatin attachment.

## 6. Outlook and Future Directions

The authors are concerned that they may have raised more questions than provided firm answers. This may be at least partly due to the small number of researcher worldwide working in this niche field that is distinct from the scientific and conceptual mainstream. We hope that we have comprehensively discussed the current state of knowledge on FV assembly and particle release and that we have provided good reasons to continue researching these and related topics. New data and insights will not only increase what is known, but may also contribute to a dynamic view on how the present has developed from the past. Here, experienced data mining, together with sophisticated concepts and tools in molecular and functional evolution, may provide testable hypotheses that can be returned to the molecular virology labs for experimental validation. The new insights may also—and this has not yet been touched on in this review—provide new ideas for translational research: a comprehensive understanding of the molecular biology, and the underlying mechanisms are needed as the basis for engineering novel vectors for gene or mRNA/miRNA transfer or vaccine antigen delivery and presentation. Outside views on the data presented and discussed here and elsewhere may drive FV research into new and exciting directions.
